# Co-Circulation of ST11-KL30 and ST11-KL64 Carbapenem-Resistant *Klebsiella pneumoniae* in Urumqi, Xinjiang, China: Molecular Epidemiology, Antimicrobial Resistance, and Virulence

**DOI:** 10.3390/pathogens15101089

**Published:** 2026-10-16

**Authors:** Hefei Zha, Qian Shi, Yongxin Li, Chunyan Liu, Xin Zhang

**Affiliations:** 1 Department of Medical Laboratory, Hospital of Xinjiang Production and Construction Corps/Second Affiliated Hospital of Shihezi University School of Medicine, Urumqi 830002, China; zha109901@126.com (H.Z.); shiqian1985101518@163.com (Q.S.); lyx_xju@sina.com (Y.L.); liuchunyan0204@126.com (C.L.); 2 Bingtuan Clinical Medical Research Center for Medical Laboratory, Urumqi 830002, China

**Keywords:** carbapenem-resistant *Klebsiella pneumoniae*, hypervirulence, molecular epidemiology, ST11 type, KL30, KL64

## Abstract

Carbapenem-resistant *Klebsiella pneumoniae* (CRKP) and hypervirulent CRKP (hv-CRKP) are growing global threats with regional heterogeneity. We collected 93 non-duplicate CRKP isolates from a hospital in Urumqi, China, from January to December 2024. Antimicrobial susceptibility was tested using the VITEK 2 system, hypermucoviscosity was assessed by the string test, resistance and virulence genes were detected by polymerase chain reaction (PCR), and clonal lineages were determined by multilocus sequence typing (MLST) and capsular typing. Isolates were mainly from the intensive care unit (ICU) (36.56%) and respiratory department (31.18%), with sputum (51.62%) the predominant specimen type. Resistance rates to most β-lactams exceeded 97%. *bla_KPC_* was the predominant carbapenemase gene (94.62%), and hv-CRKP accounted for 54.84% of isolates. Sequence type 11 (ST11) was the dominant clone (89.25%), with K-locus types (KL30) (44 isolates) and KL64 (31 isolates) most common. ST11-KL30 exhibited significantly lower resistance rates to amikacin, gentamicin, trimethoprim-sulfamethoxazole, and tigecycline, and lower *bla_TEM_* carriage compared with ST11-KL64 (*p* < 0.05); it also showed a lower proportion of hv-CRKP and string test positivity. This study is the first to show that ST11-KL30 and ST11-KL64 co-circulate as dominant hv-CRKP-carrying clones in Urumqi, clarifying their resistance and phenotypic differences and providing key data for regional surveillance.

## 1. Introduction

Carbapenem-resistant *Klebsiella pneumoniae* (CRKP) infections pose a serious threat to clinical outcomes, hospital infection control, and global public health security, with a continuously escalating epidemiological trend [1,2]. Globally, the pooled prevalence of hospital-acquired CRKP infections is approximately 28.69%, reaching as high as 66.04% in South Asia [3]. In China, the isolation rate of CRKP increased from 6.4% to 22.1% between 2014 and 2024 [4]. Of particular concern, hypervirulent CRKP (hv-CRKP), which combines carbapenem resistance with a hypervirulent phenotype, can cause severe infections and further complicate clinical management [5]. Collectively, these findings reveal the critical status of the CRKP epidemic and underscore the urgent and essential need to strengthen prevention and control measures.

Studies have shown that the most common STs among global CRKP isolates are ST11, ST258, ST147, ST15, and ST307. Among carbapenemases, KPC-type (Klebsiella pneumoniae carbapenemase) is the most prevalent, followed by NDM (New Delhi metallo-β-lactamase) and OXA-48 (oxacillinase-48) types [6]. The dissemination of CRKP exhibits marked regional and carbapenemase-type associations. Specifically, the dominant Asian clone ST11 mainly carries KPC-2 and is frequently associated with capsular types KL64, KL47, and KL25 [7,8]; the major North American and European clone ST258 predominantly possesses KL107 and often harbors KPC-2 or KPC-3 [5]; while ST147 is endemic in Europe (Italy, Greece), South Asia (India), and North Africa, with its most common capsular type being KL64 and a strong link with NDM-type enzymes [9,10].

Since its introduction into China in 2010, ST11 CRKP has gradually become the absolutely dominant clone, accounting for over 60% of domestic CRKP isolates [11]. However, driven by the convergent evolution of resistance and virulence plasmids, ST11 CRKP is now undergoing subclone replacement in China, characterized by the iterative succession of dominant clones and the spread of new high-risk clones. The isolation proportion of ST11-KL64 increased dramatically, from 1.54% in 2011 to 46.08% in 2021, progressively replacing the previously prevalent ST11-KL47 [4]. This evolution likely originated from homologous recombination in an approximately 154 kb region near the capsule synthesis and lipopolysaccharide modification loci of ST11-KL47, enabling ST11-KL64 to acquire a novel capsular type while retaining its resistance backbone [12]. Concurrently, ST11-KL64 acquired *pLVPK*- or *pK2044*-like virulence plasmids carrying virulence genes such as *rmpA*, *rmpA2*, and *iucA*, thereby evolving into hv-CRKP with both robust antimicrobial resistance and hypervirulence [13,14]. The antimicrobial resistance rates and virulence gene carriage rates of ST11-KL64 were significantly higher than those of ST11-KL47 and other non-KL64 strains, with 64.3% exhibiting a hypervirulent phenotype, making it the primary carrier of hv-CRKP in China [4]. Capsular switching in ST11 strains is not restricted to KL47 and KL64. In Aral, southern Xinjiang, a novel ST11-KL30 CRKP strain was reported, which may have evolved through the horizontal transfer of an approximately 57 kb capsular gene cluster between ST11-KL64 and an ST29-KL30 donor strain. Although this strain displayed lower virulence than ST11-KL47 and ST11-KL64, it exhibited a compensatory increase in biofilm formation capacity [15]. The subclone replacement, capsular diversity evolution, and hypervirulence differentiation observed in ST11 CRKP in China reflect the remarkable genomic plasticity of this clonal group and underscore the necessity for continuous molecular surveillance.

The dynamic evolution of ST types in CRKP reflects its adaptive evolution to environmental pressures [16]. Xinjiang, located in northwestern China, is a vast region bordering multiple countries and inhabited by diverse ethnic groups. As a major livestock production area, its complex ecological environment may facilitate unique pathways for microbial dissemination. However, in Urumqi, the economic and cultural center of Xinjiang, the molecular epidemiological characteristics of CRKP and hv-CRKP remain unexplored, and the composition and evolutionary trends of local dominant clones are still unclear. CRKP prevalence remains high both globally and within China; the novel ST11-KL30 clone reported in southern Xinjiang poses a specific evolutionary risk, and significant differences in resistance and virulence phenotypes among capsular types directly affect clinical anti-infective therapy. For these reasons, a systematic molecular epidemiological study of CRKP in the Urumqi area is of great clinical and public health significance.

## 2. Materials and Methods

### 2.1. Bacterial Isolates and Clinical Data Collection

Between January 2024 and December 2024, CRKP isolates were consecutively collected from clinical specimens of inpatients at the Xinjiang Production and Construction Corps Hospital, a tertiary teaching hospital serving as a regional medical center. Isolates were considered clinically significant pathogens based on specimen quality, specimen-appropriate semiquantitative/quantitative colony counts, and the patient’s clinical condition. With reference to the National Guide to Clinical Laboratory Procedures (4th edition) [17], the interpretation criteria for each specimen type were as follows: predominant growth of a potential pathogen or growth concordant with Gram-stain results in qualified sputum specimens, a colony count ≥ 10^4^ CFU/mL in urine, and any bacterial growth in sterile body fluids were considered clinically significant; contaminated or mixed-flora specimens were excluded. All interpretations were made in conjunction with the patient’s clinical presentation. Duplicate isolates from the same patient with the same specimen type were excluded (only the first isolate during hospitalization was retained) to avoid bias in resistance rates and typing results, ultimately yielding 93 non-duplicate CRKP isolates. Corresponding clinical data, including sex, age, ward distribution, and specimen type, were simultaneously retrieved from the hospital’s Laboratory Information System. All isolates were derived from inpatients, with a median time from admission to specimen collection of 2 days (interquartile range: 1–16 days). Of these, 49 isolates (52.7%) were collected within 48 h of admission, and 44 (47.3%) after 48 h.

### 2.2. Bacterial Isolation, Identification, and Antimicrobial Susceptibility Testing

The collected isolates were cultured overnight on blood agar plates (Zhengzhou Antu Bio, Zhengzhou, China) at 37 °C in 5% CO_2_ and identified using matrix-assisted laser desorption/ionization time-of-flight mass spectrometry (Bruker, Bremen, Germany). A bacterial suspension equivalent to a McFarland standard of 0.5 was prepared. Antimicrobial susceptibility testing (AST) was performed using the disk diffusion method (Kirby-Bauer) for amoxicillin-clavulanic acid, ceftazidime-avibactam, ceftriaxone, and gentamicin, while other antimicrobial agents were tested using N335 susceptibility cards and the VITEK 2 automated system (bioMérieux, Durham, NC, USA). Minimum inhibitory concentrations (MICs) and inhibition zone diameters were interpreted according to the 34th edition (2024) of the CLSI M100 guidelines issued by the Clinical and Laboratory Standards Institute (CLSI). *Escherichia coli* ATCC 25922 was used as the quality control strain, and all MIC values fell within the acceptable CLSI ranges. The MICs of all isolates against imipenem and meropenem were interpreted based on the 2024 CLSI criteria, and isolates resistant to at least one of these two antibiotics were defined as CRKP. MIC_50_ and MIC_90_ were defined as the MICs that inhibited the growth of 50% and 90% of the tested isolates, respectively. The results were compared with the 2024 China Bacterial Resistance Surveillance data (https://www.hnysfww.com/article.php?id=4174, accessed on 15 June 2026).

### 2.3. String Test

String tests were performed to identify the isolates with a hypermucoviscous phenotype [18]. Isolates were cultured overnight on blood agar plates at 37 °C in a 5% CO_2_ atmosphere. A single colony was gently contacted with an inoculation loop and lifted vertically upward. Formation of a viscous string > 5 mm in length upon stretching was considered a positive result, indicating a hypermucoviscous phenotype. Each isolate was tested twice, and only those with two positive results were defined as hypermucoviscous.

### 2.4. Bacterial DNA Extraction

Bacterial DNA was extracted using the standard boiling method [19]. Briefly, 1000 μL bacterial suspension was centrifuged at 12,000× *g* for 10 min, and the supernatant was discarded. The pellet was resuspended in 200 μL distilled water and heated at 100 °C for 15 min on a heating block. After cooling on ice, the lysate was centrifuged again at 12,000× *g* for 10 min. The resulting supernatant was collected and used as a template for PCR. The extracted DNA was quantified using a NanoDrop spectrophotometer (Thermo Fisher Scientific, Wilmington, DE, USA), with concentrations ranging from 80 to 100 ng/μL and A260/A280 ratios between 1.84 and 2.04, indicating sufficient purity for subsequent PCR amplification.

### 2.5. Detection of Resistance and Virulence Genes

PCR was performed to detect carbapenemase genes (*bla_KPC_*, *bla_NDM_*, *bla_IMP_*, *bla_VIM_*, and *bla_OXA-_*_48_), other β-lactamase genes (*bla_TEM_*, *bla_SHV_*, *bla_CTX-M_*, and *bla_DHA_*), outer membrane porin genes (*ompK35* and *ompK36*), efflux pump genes (*acrA*, *kexD*, *kpnE*, and *emrB*), the plasmid-mediated colistin resistance gene (*mcr-1*), and virulence genes (*rmpA*, *rmpA2*, *iucA*, *iroB*, *peg344*, *irp2*, *iutA*, and *ybts*). The primer sequences and annealing temperatures are listed in Table 1.

PCR was carried out in a 25-μL reaction mixture containing 12.5 μL of Premix Taq™ (TaKaRa Bio Inc., Kusatsu, Japan), 0.5 μL each of forward and reverse primers (10 μmol/L), and 30–50 ng of DNA template, with double-distilled water added to a final volume of 25 μL. The amplification program consisted of an initial denaturation at 95 °C for 5 min; 30 cycles of denaturation at 95 °C for 45 s, annealing at temperatures specific to each primer (Table 1) for 45 s, and extension at 72 °C for 30–120 s; followed by a final extension at 72 °C for 10 min. In each run, double-distilled water instead of the DNA template was used as a blank control, and *Escherichia coli* ATCC 25922 was used as a negative control. The amplified products were detected by 1.5% agarose gel electrophoresis, and positive products were selected for sequencing to further confirm the genes.

### 2.6. Definition of hv-CRKP

In the present study, hv-CRKP was defined as an isolate harboring *rmpA*, *rmpA2*, *iucA*, *iroB*, and *peg-344*. This definition follows the Chinese expert consensus on the diagnosis, treatment, and prevention of hypervirulent carbapenem-resistant *Klebsiella pneumoniae* infections [27] as well as related literature [28].

### 2.7. Capsular Typing

The *wzi* gene (Table 1) was amplified using PCR to determine the KL type of the isolates. The amplified products were bidirectionally sequenced by Xinjiang Youkang Biotechnology Co., Ltd. (Urumqi, China). The forward and reverse sequencing reads were assembled and manually edited to generate complete *wzi* gene sequences. The resulting sequences were submitted to the Pasteur database (https://bigsdb.pasteur.fr/klebsiella/, accessed on 28 April 2026) for comparison to determine the *wzi* allele and KL type.

### 2.8. Multilocus Sequence Typing

Multilocus sequence typing (MLST) was performed according to the scheme in the Pasteur database (https://bigsdb.pasteur.fr/klebsiella/, accessed on 28 April 2026). Seven housekeeping genes (*rpoB*, *gapA*, *mdh*, *pgi*, *phoE*, *infB*, and *tonB*) of *K. pneumoniae* were amplified using PCR with the primer sequences provided in the database. The amplified products were bidirectionally sequenced by Xinjiang Youkang Biotechnology Co., Ltd. (Urumqi, China). The resulting sequences were submitted to the same database for comparison to determine the sequence type of each isolate. A phylogenetic tree was constructed based on the concatenated sequences of the seven housekeeping genes using the maximum-likelihood method in MEGA 7.0, with branch node confidence assessed by 1000 bootstrap replicates; bootstrap values > 50% were considered to indicate acceptable confidence. The phylogenetic tree was then visualized and annotated using the online tool Chiplot (https://www.chiplot.online/, accessed on 20 June 2026).

### 2.9. Statistical Analysis

Antimicrobial susceptibility data were analyzed using WHONET 5.6 software. Statistical analyses were performed using SPSS version 25.0. Continuous variables with a non-normal distribution were expressed as medians (interquartile range [IQR]). Categorical variables are presented as numbers and percentages (*n*, %). Comparisons between groups were performed using the chi-square test or Fisher’s exact test (when the expected frequencies were <5), as appropriate. *p* < 0.05 was considered statistically significant.

## 3. Results

### 3.1. Clinical Characteristics of the Isolates

**Figure 1:**
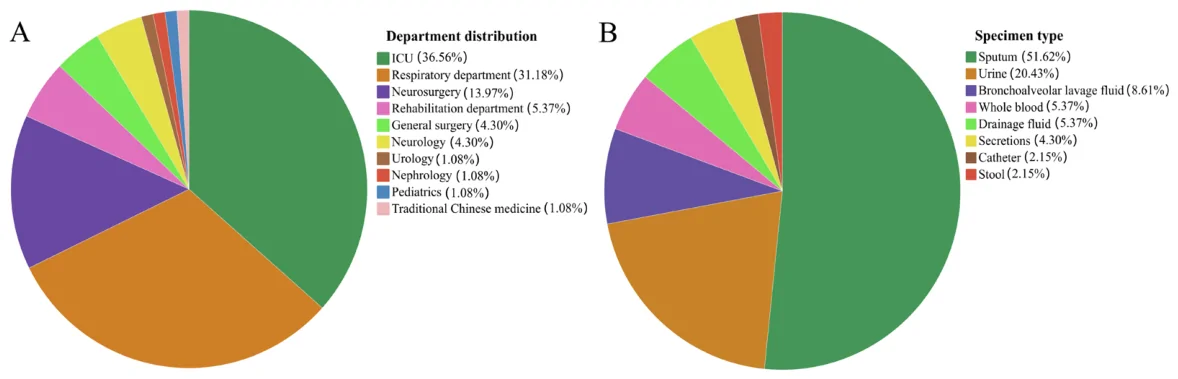
Distribution of strains by department and specimen type. (**A**) Distribution by department; (**B**) Distribution by specimen type. ICU, intensive care unit.

A total of 93 CRKP isolates were included. The median age of infected patients was 75.5 years, with 69.89% being male. Most patients were admitted to the intensive care unit (ICU, 36.56%), followed by the respiratory department (31.18%) and neurosurgery department (13.97%). Sputum (51.62%) and urine (20.43%) were the most common specimen types. Detailed clinical characteristics are presented in Figure 1.

### 3.2. Antimicrobial Susceptibility of CRKP Isolates

Antimicrobial susceptibility profiles (including MIC_50_ and MIC_90_) of the 93 CRKP isolates were analyzed and compared with 2024 national data from the CHINET surveillance program (Table 2). Resistance rates to most β-lactam antibiotics (e.g., carbapenems and third- and fourth-generation cephalosporins) exceeded 97%, with no significant differences compared with those at national levels (all *p* > 0.05). However, the resistance rate to ceftazidime/avibactam (CZA; 7.53%) was significantly lower than the national average (15.8%; *p* < 0.05). Among non-β-lactam antibiotics, the isolates showed significantly lower resistance rates to amikacin, gentamicin, and trimethoprim-sulfamethoxazole and significantly higher resistance rates to minocycline and tigecycline than those in the national data (all *p* < 0.001). No significant differences were observed in resistance rates to levofloxacin, ciprofloxacin, and colistin between the study isolates and national data (all *p* > 0.05).

### 3.3. Distribution of Carbapenemase Types, Resistance Genes, and Virulence Genes in CRKP Isolates

Among the 93 CRKP isolates, the predominant carbapenemase gene was *bla_KPC_* (94.62%), followed by *bla_NDM_* (4.30%) and *bla_IMP_* (1.08%); no *bla_VIM_* or *bla_OXA-_*_48_ was detected. The carriage rates of other β-lactamase genes were 100.00% for *bla_SHV_*, 94.62% for *bla_CTX-M_*, and 26.88% for *bla_TEM_*. *bla_DHA_* was detected in 1.08% of isolates, and no isolate carried *mcr-1*. Outer membrane protein and efflux pump-related genes were widely distributed (Table 3). Virulence gene detection rates were 89% for both *rmpA2* and *iucA*, and 55.91%, 68.82%, and 67.74% for *rmpA*, *iroB*, and *peg-344*, respectively (Table 3). Based on these results, the proportion of hv-CRKP was 54.84% (51/93).

### 3.4. Molecular Epidemiological Characteristics of CRKP Isolates

ST11 was the predominant sequence type among the 93 CRKP isolates (83/93, 89.25%), accounting for 94.32% (83/88) of KPC-producing isolates. NDM-producing isolates included ST1, ST14, ST147, and ST4661, while the IMP-producing isolate belonged to ST147. The string test positivity rate was 6.45% (6/93), and positive results were observed only among KPC-producing ST11 isolates (6/83, 7.23%). Capsular typing revealed that among KPC-producing ST11 isolates, KL30 (44/83, 53.01%) and KL64 (31/83, 37.35%) were the major capsular types; KL10 (3/83, 3.61%), KL24KL54KL55 (1/83, 1.21%), and KL47 (1/83, 1.21%) were also detected, while three isolates remained untypeable (Table 4). In addition, ST395 (3 isolates, KPC-producing) carried KL2KL30, and ST2193 (2 isolates, KPC-producing) carried KL30. Among NDM-producing isolates, ST1 carried KL109, ST14 carried KL2KL30, ST147 carried KL64, and ST4661 carried KL3; the IMP-producing isolate was ST147 (KL64). For non-ST11 isolates, the number of isolates within each ST was small, and each ST contained only one KL type; however, some KL types were shared among different STs (Table 4).

### 3.5. Phylogenetic Analysis Based on MLST

**Figure 2:**
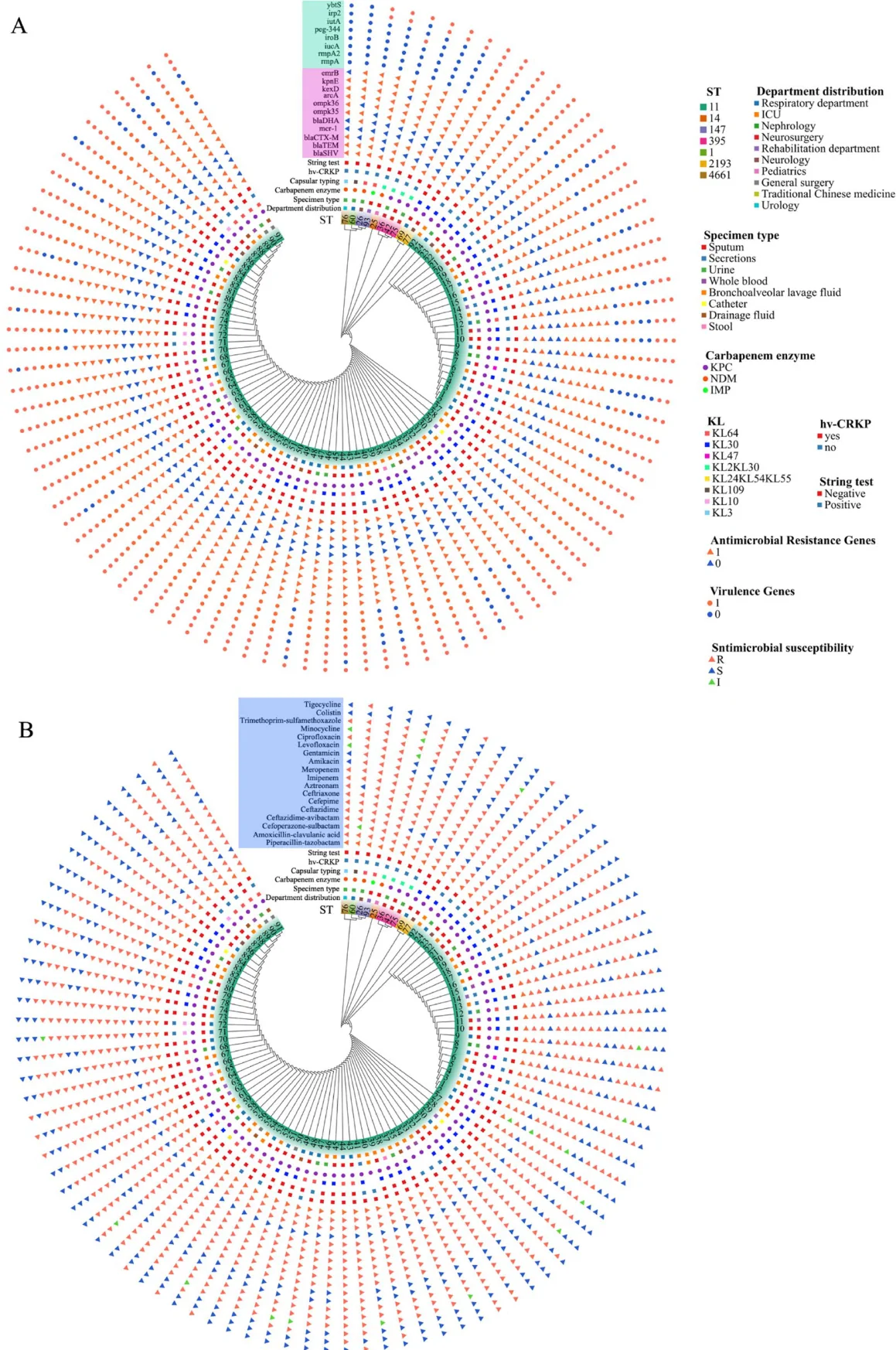
Phylogenetic tree of 93 Carbapenem-resistant *Klebsiella pneumoniae* (CRKP) isolates based on multilocus sequence typing (MLST). Maximum likelihood tree generated from MLST sequences of 93 CRKP isolates. The tree is presented in a multi-layered radial format. In Panel (**A**), the rings from the inner to the outer represent, in order, ST type, department distribution, specimen type, carbapenemase type, capsular type, hypervirulence status, string test result, antimicrobial resistance genes, and virulence genes. In Panel (**B**), the rings from the inner to the outer represent, in order, ST type, department distribution, specimen type, carbapenemase type, capsular type, hypervirulence status, string test result, and antimicrobial susceptibility. Different colors within each band distinguish the corresponding categories. “1” indicates the presence of the gene, and “0” indicates its absence.

The maximum-likelihood phylogenetic tree constructed based on MLST sequences (Figure 2) showed that the 93 CRKP isolates formed seven distinct clonal branches, displaying a co-existence pattern of dominant and sporadic clones. ST11 constituted the major clonal branch and was closely associated with capsular types KL30 and KL64, as well as the KPC-type carbapenemase. The vast majority of hv-CRKP isolates (49/51, 96.08%) and all six string-test-positive isolates were clustered within the ST11 branch. In addition, two hv-CRKP isolates belonging to ST2193-KL30 carried KPC-type carbapenemase. Isolates harboring NDM-type (ST1, ST14, ST147, and ST4661) and IMP-type (ST147) carbapenemases were scattered across different small branches without forming obvious clonal clusters, and exhibited relatively large genetic distances from the major ST11 clonal branch.

### 3.6. Comparison of Clinical and Molecular Characteristics Between ST11-KL30 and ST11-KL64 Isolates

**Figure 3:**
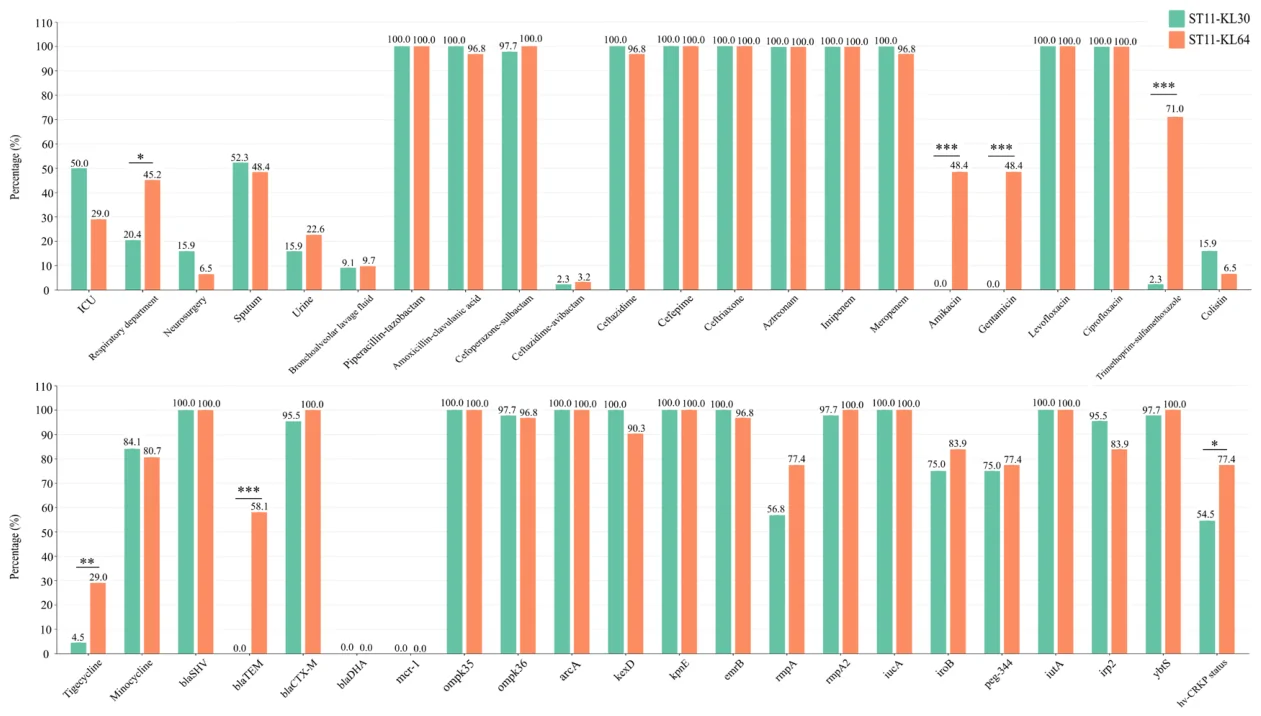
Comparison of clinical and molecular characteristics between ST11-KL30 and ST11-KL64 isolates. The comparison includes ward distribution, specimen type, antimicrobial resistance rates, resistance gene carriage rates, virulence gene carriage rates, and hypervirulence status (hv-CRKP). Asterisks indicate statistical significance: * *p* < 0.05, ** *p* < 0.01, *** *p* < 0.001. ICU, intensive care unit; CRKP, Carbapenem-resistant *Klebsiella pneumoniae*.

A comparative analysis of the two major capsular types among ST11 isolates, KL30 (*n* = 44) and KL64 (*n* = 31), was performed. The proportion of respiratory department isolates was lower for KL30 than for KL64. KL30 isolates showed significantly lower resistance rates to amikacin, gentamicin, trimethoprim-sulfamethoxazole, and tigecycline than those of KL64 isolates (all *p* < 0.05). Among resistance genes, only the detection rate of *bla_TEM_* was significantly lower in KL30 than in KL64 (*p* < 0.001). No significant differences were observed in virulence gene carriage rates between the two groups (all *p* > 0.05). However, the proportion of hv-CRKP was significantly lower in KL30 than in KL64, and the string test positivity rate among hv-CRKP isolates was also significantly lower in KL30 (0/24, 0.00%) than in KL64 (6/24, 25.00%; *p* = 0.022). Detailed results are presented in Figure 3.

## 4. Discussion

Over the past decade, the global spread of CRKP and hv-CRKP has posed a serious public health challenge. Given the marked regional heterogeneity in the molecular epidemiology of CRKP [29], elucidating region-specific epidemic patterns is essential for developing precise prevention and control strategies. In this study, we systematically characterized the epidemiological features of CRKP in Urumqi, Xinjiang, and found that KPC-producing ST11 represented the predominant epidemic clone. ST11-KL30 (53.01%) and ST11-KL64 (37.35%) were the two major circulating subtypes, with KL30 currently representing the most prevalent capsular type in this region. Furthermore, the detection rate of hv-CRKP was 54.84%, with the majority (96.08%) belonging to the ST11 clonal lineage, suggesting that ST11 is the primary clonal background associated with hv-CRKP locally. This study is the first to reveal the molecular epidemiological characteristics of CRKP in the Urumqi area of Xinjiang, and to systematically compare the resistance and molecular features between ST11-KL30 and ST11-KL64, providing critical data to support regional clonal evolution surveillance and precision-based prevention and control efforts.

In this study, the resistance rates of CRKP isolates to carbapenems and the majority of β-lactams exceeded 97%, showing no significant differences from the 2024 CHINET national surveillance data, indicating that the CRKP resistance situation in Urumqi remains severe. However, the resistance rates to certain individual agents differed significantly from the national data, warranting in-depth analysis and clinical attention. Notably, the resistance rate to CZA was significantly lower than that of the national level, suggesting that this agent holds promising application prospects for the treatment of CRKP infections in this region. The resistance rates to aminoglycosides were also relatively low, particularly pronounced among KL30-type isolates, thereby providing options for combination therapy regimens. The colistin resistance rate was similar to the national level, and no *mcr-1* gene was detected in any of the isolates, indicating that colistin remains a viable last-line therapeutic option; nevertheless, continuous surveillance of resistance evolution is warranted. In contrast, resistance rates to tigecycline and minocycline were relatively high, which may be attributable to the substantial local antimicrobial selective pressure and the high carriage rates of efflux pump genes (*acrA*, *kexD*, *kpnE*, and *emrB*) [30,31]. However, the presence of efflux pump genes does not necessarily equate to functional overexpression, and their precise roles require further validation. Additionally, a small number of isolates exhibited susceptibility or intermediate susceptibility to amoxicillin–clavulanic acid, cefoperazone–sulbactam, and ceftazidime, suggesting the existence of fine-tuned regulatory differences among isolates, such as variations in KPC hydrolytic activity, *bla_KPC_* expression levels, and synergistic deficiencies in outer membrane porins [32]. The underlying molecular mechanisms warrant further investigation.

In this study, ST11 accounted for 89.25% of the CRKP isolates, while non-ST11 isolates comprised only 10.75%, distributed among ST395, ST2193, ST147, ST14, and other types without forming secondary clonal clusters, highlighting the overwhelming predominance of ST11 in this region. Such a highly concentrated clonal distribution facilitates targeted intervention, and implies that if this clone acquires novel resistance or virulence elements, its dissemination risk would be substantially higher than that in regions with greater clonal diversity. Previous studies have shown that the capsular types of the ST11 clone in China have undergone a predominant shift from KL47 to KL64, yet the evolutionary trajectories are not entirely consistent across different regions. For instance, in Jiangxi [8] and Jiangsu [33], the proportion of KL25 strains has been steadily increasing and has become the second most prevalent type after KL64. In the present study, we found that ST11-KL30 and ST11-KL64 co-circulated in Urumqi, with KL30 being the dominant subtype. This finding is consistent with the reported prevalence of ST11-KL30 strains in Aral, southern Xinjiang [15], suggesting that, relative to the overall national trend, KL30 may possess a particular transmission advantage in the Xinjiang region. Previous studies have speculated that KL30 may have evolved from KL64 through genomic region exchange [15]. Although both belong to the same ST11 clone, KL30 isolates exhibited significantly lower resistance rates to amikacin, gentamicin, trimethoprim–sulfamethoxazole, and tigecycline compared to KL64 isolates, as well as a lower *bla_TEM_* carriage rate. This difference suggests that resistance determinants were not co-transferred or stably maintained along with the capsular gene cluster during capsular switching. It may also reflect differences in the antimicrobial selection pressures experienced by the two subtypes during their dissemination, leading to the acquisition or loss of resistance genes. In terms of ward distribution, the proportion of KL30 in the respiratory department was lower than that of KL64, but slightly higher in the ICU, suggesting that the two subtypes may have adapted to different hospital ecological niches [34,35]. The clinical implications of capsular switching warrant attention. Previous studies have shown that the 30-day all-cause mortality rate of patients infected with ST11-KL64 was significantly higher than that with ST11-KL47 [13], and the recently emerging KL25 type also exhibited enhanced fitness and virulence [8]. Therefore, although KL30 currently shows relatively higher susceptibility to certain agents, it may still acquire novel resistance elements under sustained antimicrobial selective pressure and evolve toward a more threatening direction. Continuous surveillance of its potential emergence as a new dominant clone is warranted.

hv-CRKP possesses both hypervirulence and carbapenem resistance, conferring a high risk of clinical mortality [36]. In the present study, hv-CRKP was defined according to the Chinese expert consensus and literature [28], based on the simultaneous detection of five virulence genes (*rmpA*, *rmpA2*, *iucA*, *iroB*, and *peg-344*). The detection rate was 54.84%, which is higher than rates reported in most domestic studies [37,38]. This discrepancy may be attributable to differences in the collection period and specimen source composition, suggesting a possible upward trend in hv-CRKP prevalence over time. The carriage rates of the five virulence genes were all relatively high among ST11 isolates, with *iucA* (98.80%) and *rmpA2* (97.59%) being particularly prominent, indicating that virulence plasmids harboring these genes have disseminated extensively within the ST11 clonal group. Both ST11-KL30 and ST11-KL64 served as major carriers of hv-CRKP, suggesting that the spread of hv-CRKP is driven by multiple capsular types. Surveillance strategies should therefore cover multiple types. The hypermucoviscous phenotype is one of the important virulence characteristics of hv-CRKP, mediated by the overproduction of capsular polysaccharide driven by *rmpA*/*rmpA2*, and has traditionally been observed predominantly in KL1/KL2 strains [39], with the string test being the routine assessment method. However, the hypermucoviscous phenotype of hv-CRKP does not completely align with virulence gene carriage [40]. In this study, the proportion of hypermucoviscous phenotype was 9.8%, slightly lower than the previously reported 13–22.2% [40,41]; notably, one non-hv-CRKP isolate (carrying *rmpA2*) also tested positive in the string test. Among hv-CRKP subtypes, ST11-KL30 and ST11-KL64 each accounted for 47.06%, but the string test positivity rate of KL64 was significantly higher than that of KL30, suggesting that KL64 exhibits a stronger hypermucoviscous phenotype, whereas KL30 presents as a “cryptic” hypervirulent strain. This discrepancy between genotype and phenotype may result from mutations in the *rmpA*/*rmpA2* genes leading to loss of protein function [42], or from downregulation of the hypermucoviscous phenotype to enhance colonization and environmental adaptability [43]. These hypotheses warrant further functional validation.

This study has several limitations. First, this was a single-center cross-sectional study with a limited sample size, which may affect the validity of some statistical analyses. Moreover, cross-sectional data can only capture molecular epidemiological characteristics at a specific time point, making it challenging to dynamically track clonal evolution and provide early warning of emerging resistance clones. Second, the detection of resistance genes, virulence genes, and molecular typing relied primarily on PCR-based methods. Although this approach efficiently identifies target genes, it cannot fully capture critical information such as gene sequence variations, insertion sequence structures, promoter region mutations, and gene integrity. Furthermore, while MLST can determine the sequence type of isolates, its resolution based on housekeeping gene sequences is limited, and it cannot reveal single-nucleotide polymorphism differences or fine-scale clonal evolutionary relationships within the same ST. To address these gaps, our future work will employ multi-center prospective surveillance, whole-genome sequencing and comparative genomics, targeted sequencing of regulatory genes and transcriptomics, as well as animal infection models, to systematically compensate for the current deficiencies in sample representativeness, depth of genetic information, and phenotypic validation.

## 5. Conclusions

This study reveals, for the first time, the unique molecular epidemiological landscape of CRKP in Urumqi, Xinjiang: ST11-KL30 and ST11-KL64 co-circulate, and both constitute the major types among hv-CRKP isolates; ST11-KL30 isolates exhibited lower resistance rates to multiple non-β-lactam antibiotics and a lower string test positivity rate compared with ST11-KL64 isolates. These findings underscore the importance of regional molecular epidemiological surveillance of CRKP and provide critical scientific evidence for precision-based prevention and clinical treatment in this region.

## Figures and Tables

**Table 1:** Primer sequences and amplification conditions.

Gene	Forward Primer Sequence (5′-3′)	Reverse Primer Sequence (5′-3′)	Length (bp)	Annealing Temperature (°C)
*bla_KPC_* [20]	TGTCACTGTATCGCCGTC	CAGTGCTCTACAGAAAACC	1009	52
*bla_NDM_* [20]	GCAGCTTGTCGGCCATGCGGGC	GGTCGCGAAGCTGCGCACCGCAT	782	52
*bla_IMP_* [21]	CTACCGCAGCAGAGTCTTTG	AACCAGTTTTGCCTTACCAT	587	55
*bla_VIM_* [22]	GATGGTGTTTGGTCGCATA	CGAATGCGCAGCACCAG	390	52
*bla_OXA-_*_48_ [22]	GCGTGGTTAAGGATGAACAC	CATCAAGTTCAACCCAACCG	438	57
*bla_TEM_* [23]	TCGGGGAAATGTGCG	TGCTTAATCAGTGAGGCACC	877	55
*bla_SHV_* [23]	ATGCGTTATATTCGCCTGTG	TTAGCGTTGCCAGTGCTCGATC	861	56
*bla_CTX-M_* [21]	GGTGATGAACGCTTTCCAAT	TTATCACCTACAGTCCACGA	518	62
*bla_DHA_* [23]	CTGATGAAAAAATCGTTATC	ATTCCAGTGCACTCAAAATA	898	55
*ompK35* [23]	GGATGGAAAGATGCCTTCAG	CATGACGAGGTTCCATTGTG	1392	55
*ompK36* [23]	GGGAAGAATCGCACGAAATA	TCTTACCAGGGCGACAAGAG	1744	55
*acrA* [23]	GTCCTCAGGTCAGTGGCATTA	ATTGCTCTGCTGCGCCGTT	257	55
*kexD* [23]	ACCGGTTGCGCAATACCCTGA	CGTAATTGACGCCATCCCTG	184	55
*kpnE* [23]	ATTGCTGAAATTACCGGCAC	AAATACCGATCCCTTCCCAC	172	55
*emrB* [23]	CGACATTCATGCAGGTGCT	GGTCGCCGTGTAACCGTAG	848	55
*mcr-1* [24]	AGTCCGTTTGTTCTTGTGGC	AGATCCTTGGTCTCGGCTTG	320	61
*rmpA* [25]	ACTGGGCTACCTCTGCTTCA	GCATGAGCCATCTTTCA	532	56
*rmpA2* [22]	ATTTCGGCCTAAAGCAGTTA	CTAGGTATTTGATGTGCACC	568	54
*iucA* [20]	AATCAATGGCTATTCCCGCTG	CGCTTCACTTCTTTCACTGACAGG	322	56
*iroB* [20]	ATCTCATCATCTACCCTCCGCTC	GGTTCGCCGTCGTTTTCAA	322	56
*peg-344* [22]	GCGGGAAAGGACAGAAAGCCAGTG	GAGGGAAGATGAGAAATACGAGC	332	56
*irp2* [26]	GCATTTTCCGTATCGCTCT	GCTTCATAACCTGCCTGATG	2656	56
*iutA* [25]	GGCTGGACATCATGGGAACTGG	CGTCGGGAACGGGTAGAATCG	302	66
*ybts* [20]	GACGGAAACAGCACGGTAAA	GAGCATAATAAGGCGAAAGA	242	53
*wzi* [22]	GTGCCGCGAGCGCTTTCTATCTTGGTATTCC	GAGAGCCACTGGTTCCAGAATTACCGC	580	55

**Table 2:** Antimicrobial resistance rates of CRKP isolates in this study compared with CHINET national data.

Antimicrobial Agent		MIC_50_	MIC_90_	CRKP Isolates in This Study (*n* = 93)	CHINET National Data (*n* = 14,781)	*p*
β-lactams	Piperacillin-tazobactam	≥128	≥128	93 (100.00)	98.7	0.635
	Amoxicillin-clavulanic acid	*	*	92 (98.92)	-	/
	Cefoperazone-sulbactam	≥64	≥64	91 (97.85)	94.7	0.242
	Ceftazidime-avibactam	*	*	7 (7.53)	15.8	0.029
	Ceftazidime	≥64	≥64	92 (98.92)	97.9	1.000
	Cefepime	≥32	≥64	93 (100.00)	96.2	0.051
	Ceftriaxone	*	*	93 (100.00)	98.6	0.642
	Aztreonam	≥64	≥64	92 (98.92)	-	/
	Imipenem	≥16	≥16	93 (100.00)	96.7	0.077
	Meropenem	≥16	≥16	92 (98.92)	97.3	0.522
non-β-lactams	Amikacin	≤2	≥64	20 (21.51)	67.1	<0.001
	Gentamicin	*	*	20 (21.51)	76.3	<0.001
	Levofloxacin	≥8	≥8	91 (97.85)	93.3	0.080
	Ciprofloxacin	≥4	≥4	93 (100.00)	96.6	0.078
	Minocycline	≥16	≥16	76 (81.721)	59.7	<0.001
	Trimethoprim-sulfamethoxazole	≤20	≥320	32 (34.41)	70.0	<0.001
	Colistin	≤1	8	13 (13.98)	10.5	0.277
	Tigecycline	0.5	≥8	16 (17.20)	6.9	<0.001

“*” indicates the disk diffusion method, “-” indicates no data available, and “/” indicates not applicable for statistical analysis. CRKP, Carbapenem-resistant *Klebsiella pneumoniae*.

**Table 3:** Distribution of carbapenemase types, resistance genes, and virulence genes in CRKP isolates.

Category	Gene	Number of Isolates (*n*)	Prevalence (%)
Carbapenemase genes	*bla_KPC_*	88	94.62
	*bla_NDM_*	4	4.30
	*bla_IMP_*	1	1.08
β-lactamase genes	*bla_SHV_*	93	100.00
	*bla_TEM_*	25	26.88
	*bla_CTX-M_*	88	94.62
	*bla_DHA_*	1	1.08
Colistin resistance gene	*mcr-1*	0	0.00
Outer membrane protein genes	*ompk35*	92	98.92
	*ompk36*	90	96.77
Efflux pump genes	*arcA*	93	100.00
	*kexD*	89	95.70
	*kpnE*	93	100.00
	*emrB*	91	97.85
Virulence genes	*rmpA*	52	55.91
	*rmpA2*	83	89.25
	*iucA*	84	90.32
	*iroB*	64	68.82
	*peg-344*	63	67.74
	*iutA*	84	90.32
	*irp2*	82	88.17
	*ybtS*	90	96.77

**Table 4:** Molecular Epidemiological Characteristics of Carbapenem-resistant *Klebsiella pneumoniae* Isolates.

Carbapenemase Type	MLST	String Test	Capsular Type
KPC (88)	ST11 (83)	Positive (6)	KL30 (44, 53.01%), KL64 (31, 37.35%), KL10 (3, 3.61%), KL24KL54KL55 (1, 1.21%), KL47 (1, 1.21%), - (3, 3.61%)
	ST395 (3)	Positive (0)	KL2KL30 (3, 100.00%)
	ST2193 (2)	Positive (0)	KL30 (2, 100.00%)
NDM (4)	ST1 (1)	Positive (0)	KL109 (1, 100.00%)
	ST14 (1)	Positive (0)	KL2KL30 (1, 100.00%)
	ST147 (1)	Positive (0)	KL64 (1, 100.00%)
	ST4661 (1)	Positive (0)	KL3 (1, 100.00%)
IMP (1)	ST147 (1)	Positive (0)	KL64 (1, 100.00%)

“-” indicates untypable isolates.

## Data Availability

The datasets used in this study are available from the corresponding author upon request.

## Abbreviations

The following abbreviations are used in this manuscript:

CRKP	Carbapenem-resistant *Klebsiella pneumoniae*
hv-CRKP	Hypervirulent carbapenem-resistant *Klebsiella pneumoniae*
KPC	Klebsiella pneumoniae carbapenemase
NDM	New Delhi metallo-β-lactamase
OXA-48	Oxacillinase-48
MLST	Multilocus Sequence Typing
PCR	Polymerase Chain Reaction
ICU	Intensive Care Unit
MIC	Minimum Inhibitory Concentration

## References

[1] Li P., Luo W.Y., Xiang T.X., Peng T.X., Luo S., He Z.Y., Liao W., Wei D.D., Liu P., Wan L.G. (2023). Isolation of Hv-CRKP with co-production of three carbapenemases (blaKPC, blaOXA-181 or OXA-232, and blaNDM-1) and a virulence plasmid: A study from a Chinese tertiary hospital. Front. Microbiol..

[2] Jin X., Chen Q., Shen F., Jiang Y., Wu X., Hua X., Fu Y., Yu Y. (2021). Resistance evolution of hypervirulent carbapenem-resistant *Klebsiella pneumoniae* ST11 during treatment with tigecycline and polymyxin. Emerg. Microbes Infect..

[3] Lin X.C., Li C.L., Zhang S.Y., Yang X.F., Jiang M. (2023). The Global and Regional Prevalence of Hospital-Acquired Carbapenem-Resistant *Klebsiella pneumoniae* Infection: A Systematic Review and Meta-analysis. Open Forum Infect. Dis..

[4] Wang Q., Wang R., Wang S., Zhang A., Duan Q., Sun S., Jin L., Wang X., Zhang Y., Wang C. (2024). Expansion and transmission dynamics of high risk carbapenem-resistant *Klebsiella pneumoniae* subclones in China: An epidemiological, spatial, genomic analysis. Drug Resist. Updat..

[5] Budia-Silva M., Kostyanev T., Ayala-Montaño S., Bravo-Ferrer Acosta J., Garcia-Castillo M., Cantón R., Goossens H., Rodriguez-Baño J., Grundmann H., Reuter S. (2024). International and regional spread of carbapenem-resistant *Klebsiella pneumoniae* in Europe. Nat. Commun..

[6] Shi Y., Ru D., Wang M., Cao S., Chen D., Si Z., Zhang Y., Li Y., Lu J. (2026). Global genomic epidemiology of carbapenemase-producing *Klebsiella pneumoniae*, 1996–2023. Int. J. Antimicrob. Agents.

[7] Zhang F., Liu X., Li Z., Li Z., Lei Z., Fan Y., Yang X., Liu Q., Ma Y., Lu B. (2025). Tracking international and regional dissemination of the KPC/NDM co-producing *Klebsiella pneumoniae*. Nat. Commun..

[8] Li P., Xiao Y., Luo R., Zhang J., Nie F., Zou T., Liu Y., Zhang W., Xiang T. (2026). Comparative genomic, phenotypic, and clinical characterization of ST11-KL25 and ST11-KL64 hypervirulent carbapenem-resistant *Klebsiella pneumoniae* in a tertiary hospital, 2020-2023. Antimicrob. Agents Chemother..

[9] Jiang J., Cienfuegos-Gallet A.V., Long T., Peirano G., Chu T., Pitout J.D.D., Kreiswirth B.N., Chen L. (2025). Intricate interplay of CRISPR-Cas systems, anti-CRISPR proteins, and antimicrobial resistance genes in a globally successful multi-drug resistant *Klebsiella pneumoniae* clone. Genome Med..

[10] Peirano G., Chen L., Kreiswirth B.N., Pitout J.D.D. (2020). Emerging Antimicrobial-Resistant High-Risk *Klebsiella pneumoniae* Clones ST307 and ST147. Antimicrob. Agents Chemother..

[11] Li X., Ke H., Wu W., Tu Y., Zhou H., Yu Y. (2021). Molecular mechanisms driving the in vivo development of KPC-71-Mediated resistance to ceftazidime-avibactam during treatment of carbapenem-resistant *Klebsiella pneumoniae* infections. mSphere.

[12] Chen T., Wang Y., Zhou Y., Zhou W., Chi X., Shen P., Zheng B., Xiao Y. (2023). Recombination Drives Evolution of Carbapenem-Resistant *Klebsiella pneumoniae* Sequence Type 11 KL47 to KL64 in China. Microbiol. Spectr..

[13] Zhou K., Xiao T., David S., Wang Q., Zhou Y., Guo L., Aanensen D., Holt K.E., Thomson N.R., Grundmann H. (2020). Novel Subclone of Carbapenem-Resistant *Klebsiella pneumoniae* Sequence Type 11 with Enhanced Virulence and Transmissibility, China. Emerg. Infect. Dis..

[14] Wang R., Zhang A., Sun S., Yin G., Wu X., Ding Q., Wang Q., Chen F., Wang S., van Dorp L. (2024). Increase in antioxidant capacity associated with the successful subclone of hypervirulent carbapenem-resistant *Klebsiella pneumoniae* ST11-KL64. Nat. Commun..

[15] Zhao F., Hou X., Sun G., Fu Y., Wang L., Yao B., Liu X., Weng R., Meng Y., Zhou J. (2024). The prevalence of carbapenem-resistant Enterobacterales and the emergence of novel ST11-KL30 carbapenem-resistant *Klebsiella pneumoniae* in Xinjiang, China. J. Glob. Antimicrob. Resist..

[16] Wang S., Ma S., Sun S., Wang Q., Ding Q., Jin L., Chen F., Yin G., Wu X., Wang R. (2024). Global evolutionary dynamics of virulence genes in ST11-KL47 carbapenem-resistant *Klebsiella pneumoniae*. Int. J. Antimicrob. Agents.

[17] Shang H., Wang Y.S., Shen Z.Y. (2015). National Guide to Clinical Laboratory Procedures.

[18] Li L., Li S., Wei X., Lu Z., Qin X., Li M. (2023). Infection with carbapenem-resistant Hypervirulent *Klebsiella pneumoniae*: Clinical, virulence and molecular epidemiological characteristics. Antimicrob. Resist. Infect. Control.

[19] Tantisiriwat W., Buppanharun J., Ekpanyaskul C., Onruang K., Yungyuen T., Kiratisin P., Santiwatanakul S. (2022). In Vitro Activity of Ceftolozane-Tazobactam and Other Antibiotics against *Pseudomonas aeruginosa* Infection-Isolates from an Academic Medical Center in Thailand. Antibiotics.

[20] Wang X., Wang J., Jiang X., Huang Z., Huang L., Wei Q., Zhang L. (2025). Molecular epidemiological analysis and research on resistance and virulence of carbapenem-resistant *Klebsiella pneumoniae* in a tertiary hospital from 2016 to 2023. BMC Microbiol..

[21] Li Z., Ding Z., Yang J., Liu Y., Jin X., Xie J., Li T., Ding Y., Zeng Z., Liu J. (2021). Carbapenem-resistant *Klebsiella pneumoniae* in Southwest China: Molecular characteristics and risk factors caused by KPC and NDM producers. Infect. Drug Resist..

[22] Hu F., Pan Y., Li H., Han R., Liu X., Ma R., Wu Y., Lun H., Qin X., Li J. (2024). Carbapenem-resistant *Klebsiella pneumoniae* capsular types, antibiotic resistance and virulence factors in China: A longitudinal, multi-centre study. Nat. Microbiol..

[23] Qiu X., Jiang M., Xu J., Wu Q., Lin C., Li W., Li Q. (2025). Molecular characterization of carbapenem resistance mechanisms and phenotypic correlations in clinical *Klebsiella pneumoniae* isolates from Ningbo, China. Front. Microbiol..

[24] Aslam B., Siddique M.H., Siddique A.B., Shafique M., Muzammil S., Khurshid M., Rasool M.H., Ahmad M., Chaudhry T.H., Amir A. (2022). Distribution of mcr-1 harboring hypervirulent *Klebsiella pneumoniae* in clinical specimens and lytic activity of bacteriophage KpnM against isolates. Infect. Drug Resist..

[25] Candan E.D., Aksöz N. (2015). *Klebsiella pneumoniae*: Characteristics of carbapenem resistance and virulence factors. Acta Biochim. Pol..

[26] Russo T.A., Olson R., MacDonald U., Beanan J., Davidson B.A. (2015). Aerobactin, but not yersiniabactin, salmochelin, or enterobactin, enables the growth/survival of hypervirulent (hypermucoviscous) *Klebsiella pneumoniae* ex vivo and in vivo. Infect. Immun..

[27] Chinese Society of Infectious Disease Physicians, Chinese Medical Doctor Association, Hospital Infection Control Branch of Chinese Preventive Medicine Association, Chinese Hospital Association Clinical Microbiology Laboratory Specialized Committee, Society of Bacterial Infection and Resistance of Chinese Medical Association, Bacteriology and Mycology Group, Society of Infectious Diseases, Chinese Medical Association, National Medical Center for Infectious Diseases (Hangzhou), National Clinical Research Center for Infectious Diseases (Hangzhou), Infectious Diseases Physicians Branch of Zhejiang Medical Doctors Association, Antimicrobial Resistance and Control Committee of Zhejiang Pharmaceutical Association (2025). Expert consensus on the Diagnosis, Treatment, and Prevention of Hypervirulent Carbapenem-Resistant *Klebsiella pneumoniae* Infection. Chin. J. Infect. Control.

[28] Tang Y., Du P., Du C., Yang P., Shen N., Russo T.A., Liu C. (2025). Genomically defined hypervirulent *Klebsiella pneumoniae* contributed to early-onset increased mortality. Nat. Commun..

[29] Hu Y., Liu C., Shen Z., Zhou H., Cao J., Chen S., Lv H., Zhou M., Wang Q., Sun L. (2020). Prevalence, risk factors and molecular epidemiology of carbapenem-resistant *Klebsiella pneumoniae* in patients from Zhejiang, China, 2008–2018. Emerg. Microbes Infect..

[30] Luo X., Mu K., Zhao Y., Zhang J., Qu Y., Hu D., Jia Y., Dai P., Weng J., Wang D. (2022). Emergence of blaNDM- 1-carrying *Aeromonas caviae* K433 isolated from patient with community-acquired pneumonia. Front. Microbiol..

[31] Bharatham N., Bhowmik P., Aoki M., Okada U., Sharma S., Yamashita E., Shanbhag A.P., Rajagopal S., Thomas T., Sarma M. (2021). Structure and function relationship of OqxB efflux pump from *Klebsiella pneumoniae*. Nat. Commun..

[32] Coppi M., Di Pilato V., Monaco F., Giani T., Conaldi P.G., Rossolini G.M. (2020). Ceftazidime-Avibactam Resistance Associated with Increased *bla*_KPC-3_ Gene Copy Number Mediated by pKpQIL Plasmid Derivatives in Sequence Type 258 *Klebsiella pneumoniae*. Antimicrob. Agents Chemother..

[33] Lian J., Li Q., Peng C., Lin T., Du H., Tang C., Zhang X. (2025). Molecular and epidemiological characterization of carbapenem-resistant hypervirulent *Klebsiella pneumoniae* in Huaian, China (2022–2024): A retrospective study. Front. Cell. Infect. Microbiol..

[34] De Koster S., Rodriguez Ruiz J.P., Rajakani S.G., Lammens C., Glupczynski Y., Goossens H., Xavier B.B. (2022). Diversity in the characteristics of *Klebsiella pneumoniae* ST101 of human, environmental, and animal origin. Front. Microbiol..

[35] Chen F., Lv T., Xiao Y., Chen A., Xiao Y., Chen Y. (2021). Clinical characteristics of patients and whole genome sequencing-based surveillance of *Escherichia coli* community-onset bloodstream infections at a Non-tertiary Hospital in China. Front. Microbiol..

[36] Wang M., Tian Y., Xu L., Zhang F., Lu H., Li M., Li B. (2022). High osmotic stress increases OmpK36 expression through the regulation of KbvR to decrease the antimicrobial resistance of *Klebsiella pneumoniae*. Microbiol. Spectr..

[37] Guo M.-Q., Wang Y.-T., Wang S.-S., Chen L.-K., Xu Y.-H., Li G. (2024). Genomic epidemiology of hypervirulent carbapenem-resistant *Klebsiella pneumoniae* at Jinshan local hospital, Shanghai, during 2014-2018. J. Microbiol. Immunol. Infect..

[38] Jiang Y., Kong N., Li Z., Wu W., Xie Y., Zeng Z., Peng T., Shen C., Ouyang S. (2026). Epidemiological and characteristic differences of hypervirulent and classical *Klebsiella pneumoniae*: A clinical and genomic study in Southern China during the COVID-19 pandemic. Front. Cell. Infect. Microbiol..

[39] Catalán-Nájera J.C., Garza-Ramos U., Barrios-Camacho H. (2017). Hypervirulence and hypermucoviscosity: Two different but complementary *Klebsiella* spp. phenotypes?. Virulence.

[40] Yang X., Sun Q., Li J., Jiang Y., Li Y., Lin J., Chen K., Chan E.W.C., Zhang R., Chen S. (2022). Molecular epidemiology of carbapenem-resistant hypervirulent *Klebsiella pneumoniae* in China. Emerg. Microbes Infect..

[41] Chen H., Li T., Huang X., He Q. (2025). Emergence of ST11-KL64 carbapenem-resistant hypervirulent *Klebsiella pneumoniae* isolates harboring blaKPC-2 and iucA from a tertiary teaching hospital in Western China. BMC Infect. Dis..

[42] Shankar C., Vasudevan K., Jacob J.J., Baker S., Isaac B.J., Neeravi A.R., Sethuvel D.P.M., George B., Veeraraghavan B. (2022). Hybrid plasmids encoding antimicrobial resistance and virulence traits among hypervirulent *Klebsiella pneumoniae* ST2096 in India. Front. Cell. Infect. Microbiol..

[43] Song S., Yang S., Zheng R., Yin D., Cao Y., Wang Y., Qiao L., Bai R., Wang S., Yin W. (2024). Adaptive evolution of carbapenem-resistant hypervirulent *Klebsiella pneumoniae* in the urinary tract of a single patient. Proc. Natl. Acad. Sci. USA.

